# Annotations

**Published:** 1904-12-03

**Authors:** 


### ANNOTATIONS.

### "Procedure in Optology."

In a recent issue we expressed the hope that the anticipated permission for the "Society of Chemist-Opticians" to meet in the official premises of the Pharmaceutical Society to hear a lecture on "The Use of the Ophthalmoscope and the Retinoscope in Detecting Diseases of the Eye" would not be granted. It now appears that, for some reason or other, it was deemed well to substitute for the original title of the lecture the phrase which stands at the head of this note, and that, after this sacrifice, the Society of Chemist-Opticians, having obtained the necessary consent, met on November 21st in the Pharmaceutical Society's House to hear a lecture by a Mr. Alfred Clarke on "Procedure in Optology." Whatever may have been the original intention, the lecture, be it observed, was *not* to be on the use of the ophthalmoscope and retinoscope in detecting diseases of the eye, and the *Pharmaceutical Journal* denies indignantly that permission for a lecture dealing with these subjects had been granted, or that it had ever "hoped" for such permission. Yet when we turn to the summary of the lecture on "Procedure in Optology" as presented in the columns of that journal, we read that the lecturer "proceeded to show . . . how errors of refraction can be detected, and explained the use of the Geneva retinoscope and the ophthalmoscope." And the object of all this, we are told, is "to enable the optician to recognise cases which require treatment by an oculist from simple cases of defective sight." Whether men ignorant of anatomy, physiology, and pathology, and devoid of clinical experience can, with advantage to the public, be encouraged to profess their ability to make such distinctions (even granting that these exist) is not now under discussion. The fact to be observed is that the Pharmaceutical Society has afforded some degree of official recognition to the chemist-optician and to his "procedure in optology." If that is to be the policy of the society it would be well to have it frankly announced. Ingenious ambiguities may be left to the framers of phrases and to their journalistic accomplices.

### Tattooing and Disease.

There are several interesting facts in the annual report of the Director-General of the Medical Department of the Navy, which was issued on Monday. The aggregate number of cases of disease and injury recorded last year furnished a ratio of 831·57 per 1,000, a decrease of 29·56 per 1,000, compared with the ratio of 1902, and a decrease of 48·06 contrasted with the average of the last six years. The invaliding ratio showed a decrease of 5·93 per 1,000, and the death-rate was the lowest recorded since 1856. As to the causes of death, there was only one from a wound in action, and none of the ten cases of plague on the China Station was fatal. The number of cases of wounds and injuries was 17,295 as compared with 16,389 in the previous year, but the deaths were nearly 100 less. The Mediterranean Station shows the lowest sick-rate and the irregular force the highest. No fewer than 16 committed suicide. A notable contribution to the report is supplied by the staff surgeon of his Majesty's ship *Thetis*, who deals with the communication of disease by tattooing. He states that a stoker who came under his observation complained of a rash on his body. Upon inquiry, he ascertained that the man had been tattooed at Wei-hai-wei, and it was in the midst of a patch of tattooed skin that a sore appeared. This discovery prompted him to suggest that, as tattooing is now not uncommon in many widely separated classes of society, it would repay any one "desiring that form of decoration" to see that the needles used are sterilised, as a precaution against the many diseases that might otherwise be introduced into the system. Bearing in mind the number of persons who are believed to earn a living by tattooing, this warning as to the possibility of one of the crazes of the moment being a means of conveying loathsome maladies is not unnecessary. Unless care is exercised, the incomprehensible fascination which tattooing has for some otherwise sane English men and women cannot be yielded to without running a serious risk. The advice which has been given for the special protection of the blue-jackets we accordingly emphasise for the benefit of all members of the community who derive pleasure from having their bodies decorated, whether with birds or fishes or creeping things.

### Smoke and Fog.

The problem of fog, so far as London is concerned, has a severely practical aspect. In addition to much personal inconvenience and prejudice to the health of the citizens, the dislocation it produces in business arrangements means heavy financial loss. Recent experiences carry a somewhat acute suggestion that the comparative immunity from serious fogs enjoyed by London of recent years is about to be interrupted. But it is to be hoped that this is a false alarm, and fortunately there are substantial grounds for this anticipation. There is no doubt that for some years the density of London fogs has been steadily decreasing and it is certain that this is not some mere freak of nature, but is the result of definite and fairly well understood causes. Thanks to the provisions of the Smoke Abatement Act and to the vigour with which they have been applied by the London County Council, there has been considerable diminution in the supply of smoke, which determines the occurrence of fog in its worst form. It is the solid particles in such smoke which are responsible for this variety of atmosphere, and inasmuch as the law prohibits the production of black smoke, the way to the suppression of the extreme terrors of fog would seem to be a fairly direct one. Proverbially, however, an ideal is difficult of attainment, and though much has been accomplished more yet remains to be done. In particular the private householder continues to add to the atmosphere particles of unconsumed carbon, and though his individual contribution is small the total sum produced by the class is enormous. Another considerable source of black smoke is the railways, and there seems reason to believe that some of the companies find it more economical to pay repeated fines than to improve the quality of the coal consumed in their engines. It is understood that a committee of the Royal Society has been studying the fog question, and if science can produce some successful practical solution it will add still another item to the benefits it has conferred on mankind.

